# Studying Different Tasks of Implicit Learning across Multiple Test Sessions Conducted on the Web

**DOI:** 10.3389/fpsyg.2016.00808

**Published:** 2016-06-07

**Authors:** Werner Sævland, Elisabeth Norman

**Affiliations:** Department of Psychosocial Science, Faculty of Psychology, University of BergenBergen, Norway

**Keywords:** implicit learning, multiple sessions, time, web-based, online, ASRT, dynamic system control, DSC

## Abstract

Implicit learning is usually studied through individual performance on a single task, with the most common tasks being the Serial Reaction Time (SRT) task, the Dynamic System Control (DSC) task, and Artificial Grammar Learning (AGL). Few attempts have been made to compare performance across different implicit learning tasks within the same study. The current study was designed to explore the relationship between performance on the DSC Sugar factory task and the Alternating Serial Reaction Time (ASRT) task. We also addressed another limitation of traditional implicit learning experiments, namely that implicit learning is usually studied in laboratory settings over a restricted time span lasting for less than an hour. In everyday situations, implicit learning is assumed to involve a gradual accumulation of knowledge across several learning episodes over a longer time span. One way to increase the ecological validity of implicit learning experiments could be to present the learning material repeatedly across shorter test sessions. This can most easily be done by using a web-based setup in which participants can access the material from home. We therefore created an online web-based system for measuring implicit learning that could be administered in either single or multiple sessions. Participants (*n* = 66) were assigned to either a single session or a multiple session condition. Learning occurred on both tasks, and awareness measures suggested that acquired knowledge was not fully conscious on either of the tasks. Learning and the degree of conscious awareness of the learned regularities were compared across conditions and tasks. On the DSC task, performance was not affected by whether learning had taken place in one or over multiple sessions. On the ASRT task, RT improvement across blocks was larger in the multiple-session condition. Learning in the two tasks was not related.

## 1. Introduction

Implicit learning can broadly be defined as learning that occurs without full conscious awareness of the regularities contained in the learned material itself and/or that learning has occurred (Berry and Dienes, 1993). Moreover, the process of implicit learning is described as incidental and automatic, and not depending on explicit hypothesis testing (Seger, 1994). Several different methods for measuring implicit learning have been developed, and though quite diverse in terms of both material to be learned and means of measuring learning, they all share some underlying features with respect to their design and measurement.

### 1.1. Experimental paradigms for studying implicit learning

Cleeremans et al. (1998) described the common structure of paradigms for studying implicit learning as involving three components. First, participants are exposed to a complex rule-governed regularity where learning is incidental. Second, they perform a task which includes a measure that can quantify the degree to which the regularity has been learned. And third, the extent to which the participants are consciously aware of the knowledge learned is measured. The most common experimental paradigms used to study implicit learning are: (1) The AGL task (Reber, 1967), (2) The SRT task (Nissen and Bullemer, 1987), and (3) The DSC task (Berry and Broadbent, 1984). Though similar in principal structure, the experimental setup, manipulations, and measures used to infer implicit learning differ across these paradigms. We now briefly present the SRT and DSC tasks, since varieties of these two tasks were applied in the current study.

#### 1.1.1. Serial reaction time tasks

In SRT tasks, participants are usually shown an array of four symbols on screen, where the task is to press predefined buttons on the keyboard corresponding to the position of the symbols each time one is highlighted. When a button with a highlighted symbol has been pressed, a new trial begins. The instructions usually encourage both speed and precision. The seemingly random positions of the stimuli follows a spatial pattern that is repeated (Nissen and Bullemer, 1987). Implicit learning is commonly inferred from differences in reaction time (RT) on blocks of random trials against blocks following the rule-governed pattern, when the person shows a concurrent lack of conscious awareness of the learned sequence. Consciousness of acquired knowledge can be measured through verbal reports (Curran and Keele, 1993), and forced choice tests of recognition (Destrebecqz and Cleeremans, 2001; Schumacher and Schwarb, 2009), generation tasks of inclusion and exclusion (Destrebecqz and Cleeremans, 2001), and confidence ratings (Smith and McDowall, 2004).

A variation of the original SRT paradigm is the Alternating Serial Reaction Time (ASRT) task created by Howard and Howard (1997). The difference between traditional SRT tasks and the ASRT task is the ratio of trials on which the target follows the sequence regularity. In the SRT task, the target follows the sequence on all (i.e., deterministic sequence) or on the majority (i.e., probabilistic sequence) of trials. In the ASRT task, the target alternates between following a pre-defined sequence and random positions on every second trial. The overall regularity is thus harder to detect explicitly. As in the traditional SRT task, the main dependent variables in the ASRT task are accuracy and RT.

#### 1.1.2. Dynamic systems control tasks

In DSC tasks, participants are required to control a dynamic system and achieve a target output value through adjusting an input value in a situation where the relationship between input and output is governed by an algorithm. The first DSC task created was the sugar factory task by Berry and Broadbent (1984). In this task, participants were instructed to control the amount of workers in a simulated sugar factory and to aim for a given target production level of 9000 tons of sugar on each trial. Sugar production is a function of the previous trials sugar production, current worker input, and a small random error, but the relationship is not revealed to the participants. Learning is measured as the proportion of trials on which the output is within target limits, usually coded as +∕−1000 of target production level. The extent to which learning is conscious is measured by questioning about strategy (Witt et al., 2006), open questions or multiple choice predictions (Berry and Broadbent, 1984). The method of measuring implicit learning in DSC tasks does not rely on efficacy as there is no time limit, but on accuracy alone.

### 1.2. Theoretical questions and methodological possibilities

The current study had multiple aims. The main aims were to compare implicit learning across different tasks and study implicit learning over time across multiple test sessions. In order to enable this, we designed a set of tasks that could be administered online. Thus, a secondary aim was methodological, i.e., to address whether and how implicit learning can be studied in a web-based setting.

#### 1.2.1. Comparing implicit learning across different tasks

Implicit learning has been studied with a variety of different tasks, and there is also a diversity in the type of measures that have been developed to assess learning and awareness in different implicit learning experiments. Combined with the fact that tasks are usually studied independently, this leaves the question of whether different implicit learning tasks measure the same construct or different varieties of what is commonly referred to as implicit learning. One way to address this question is by directly comparing performance across different implicit learning tasks for the same individuals within the same experimental setting. To the authors' knowledge, only one study to date has correlated scores on tasks of AGL, SRT, and DSC (Gebauer and Mackintosh, 2007). In this experiment, the measure of learning in the SRT task was the difference in reaction time between the last random block and the preceding pattern block. The measure for the DSC was the number of trials on target. Gebauer and Mackintosh found virtually no correlation between the implicit learning tasks when conducted with typical incidental learning instructions. However, it could be argued that even though DSC tasks and typical SRT tasks both involve implicit learning, an important difference between them is that SRT learning involves the execution of a series of motor responses that correspond to the perceptual sequence. In this situation, RT performance may be facilitated by motor learning Goschke (1998), as well as the learning of lower-order frequencies of simple sequence elements Frensch and Miner (1995)^1^. One could argue that learning in ASRT tasks involve more abstract and higher order mental representations than SRT learning, which may make a comparison between ASRT and DSC learning less likely to be confounded by such influences. Therefore, in this study, we wanted to test if a relationship between scores on the ASRT task and the sugar factory task could be identified. If performance on the DSC task was not related to performance on the ASRT task, this would corroborate the conclusions by Gebauer and Mackintosh that different implicit learning tasks reflect a unitary ability.

It should be noted that even though our main reason for comparing performance across tasks was to address whether an ASRT and a DSC task involve the same cognitive processes, the comparison is also relevant to the question of whether individual differences play a role in implicit learning. This is because it could be argued that tasks of implicit learning rely on the implied assumption of both (1) a reliably constant baseline level of performance for each participant, and (2) a reliably asymptotic individual improvement in performance with practice. The reliability of implicit learning performance could in theory be explained as either indicative of dispositional traits in the participants (see Kaufman et al., 2010), or as a situationally stable combination of several factors of both the individual and the contextual demands. Irrespective of whether or not implicit learning should be regarded as a dispositional trait or as performance affected by stable situational factors, a common prerequisite for both is that of concurrent validity. If the two task were found to correlate, this could provide a starting point for further investigations into the question of whether implicit learning could be regarded as a trait that is stable in individuals across time.

#### 1.2.2. Studying the development of implicit learning across time

Another aim was to address the extent to which implicit learning is influenced by whether learning takes place during a single test session vs. across multiple test sessions. It is reasonable to assume that implicit learning in real-life settings occurs in a gradual and progressive manner over a longer time span (Norman and Price, 2012). If implicit learning was studied across a prolonged period of exposure, this could therefore be argued to increase ecological validity and the generalizability of findings to real-life settings.

To study implicit learning across time, it would be necessary to administer the tasks over several sessions spanning over multiple days. There are several examples of implicit learning experiments that have included multiple training sessions. However, the reason for administering the tasks over multiple sessions have mainly been to study implicit learning after extensive practice (Cleeremans and McClelland, 1991; Howard and Howard, 1997), higher order learning (Howard et al., 2004), and the effect of concurrent verbalization (Stanley et al., 1989). Therefore, whether or not administering implicit learning tasks over several sessions might influence learning and awareness differently from that of single session administration, still remains unclear.

Another reason for studying implicit learning across multiple test sessions administered over several days, is to reduce possible fatigue effects. Implicit learning experiments may be both monotonous and time demanding, which in combination may prevent participants from doing their best. For this reason, one may hypothesize that splitting implicit learning tasks into several sessions could potentially improve performance.

To explore how implicit learning developed across test sessions, participants were assigned to either a single session condition or a multiple session condition. In the multiple session condition the same amount of task exposure as in the single session condition was distributed over five sessions that each took place on consecutive days.

It should be noted that even though intermittent task exposure has the potential benefits accounted for above, there are also some potential concerns. For example, more factors are outside of the experimenter's control than in the case of single-session experiments. These include variation across exposure situations across test sessions, as well as possible fatigue associated with taking part across several days. Performance may also be influenced by individual differences in circadian rythm, which was not controlled for in the current experiment.

#### 1.2.3. Online assessment of implicit learning

As pointed out earlier, the tasks used to study implicit learning are often monotonous and repetitive. Anecdotal evidence from our own lab indicates that tasks are often perceived as abstract, confusing, and non-engaging. SRT tasks are dependent on reaction time as a proximate for measuring changes in learning, and when both engagement and motivation to perform is low, an increase in both variance and overall reaction time is to be expected (Weiss, 1965; Firestone and Douglas, 1975). A lack of attention might also reduce implicit learning of regularities (Nissen and Bullemer, 1987; Jiang and Chun, 2001). Creating tasks which can be experienced as motivating and engaging for participants might therefore increase the sensitivity of reaction time as a measure of learning in the ASRT task.

Typically, DSC tasks are administered without any time constraints, either overall or on individual trials. It is nevertheless possible that in the context of typical laboratory administration, participants might experience a certain time pressure. One can argue that both social comparison with other concurrent participants, and not wanting to take up more time than necessary from the experimenter might motivate participants to reduce time usage. Mann and Tan (1993) found that solving a dilemma under perceived time pressure, as induced by instructing participants to hurry their decision, reduced the amount of proposed alternatives to solve the dilemma, along with less cost/benefit reflections for each alternative. Studies on decision making under time pressure generally show detrimental effects on problem solving as opposed to decision making without a time limit (for a review of the literature, see Edland and Svenson, 1993). Tasks such as the sugar factory task might thus also benefit from online administration, where the participant would be able to perform the task at home in the absence of social pressures.

To conduct the study in a way that could easily be administered over several sessions, while also being experienced as more engaging and with the additional benefit of being less time consuming for the participants, we developed an online set of tasks that participants could take part in from their own home. Experiments using time-sensitive measures as reaction time online and in self selected settings have previously shown results comparable to those obtained in laboratory settings (Reimers and Stewart, 2007).

Research employing online assessment with other tasks than those of implicit learning have also revealed unexpected results, different from what laboratory results show. For example, Rueckert (2005) found that online assessment, as compared to traditional laboratory assessment, led to significantly greater leftward bias for right handed individuals when perceiving chimeric faces. Others, such as Houben and Wiers (2008), found that online assessment led to a stronger relationship between performance on an implicit association test and explicit measures, which may also support the notion of a relative reduction in perceived social pressures. Online testing also has several advantages for the researcher as compared to laboratory experiments, such as costs saving and a larger sampling frame (for an overview of advantages and disadvantages, see Reips, 2000).

To summarize, the research questions of the current study were

1. Is there a relationship between individual implicit learning on the DSC task and ASRT task?
2. Will learning and awareness in a DSC and ASRT task be influenced by whether exposure is distributed across multiple test sessions as compared to traditional single session administration?
3. Is it feasible to administer test sessions of implicit learning online?

## 2. Methods

### 2.1. Participants

Sixty-six participants, 14 male and 52 female, aged 19–28 (*M* = 22.09), completed the study. The majority of participants were current or former students at the University of Bergen. On completion of the test, participants were compensated in the form of a gift-card to a local bakery. Due to the added inconvenience of participating over several days for participants in the multiple sessions condition, the monetary value of the gift-card should also reflect this. Multiple session participants received a gift card valued 250 NOK, while single session participant received a gift card valued 150 NOK.

### 2.2. Design

Participants were recruited to either a Single session condition or a Multiple sessions condition. Those who were assigned to the Multiple session condition were required to partake in the study for five successive days. The Single session condition was administered in a single sitting. The conditions were equal in total amount of exposure to both tasks, and differed only in recruitment description, monetary value of gift-card, and the number of days they lasted.

The total duration of the study and each task depended on the speed of each participant, and the mean duration of both tasks is summarized in the results section.

For the sugar factory task, the design was a 2 × 5 (Session type × Block) mixed factorial, with session type (single vs. multiple) as a between-subjects variable and block (1−5) as a within-subjects variable. Dependent variables were number of Trials on target (production within target limits) and Mean confidence (1−6, from highly inconfident to highly confident). Trials on target were also dichotomized and included as a within-subject variable for the analysis of Mean confidence.

For the ASRT task, we applied a 2 × 2 × 10 (Session type × Trial type × Block) mixed factorial design, with session type (single vs. multiple) as between-subjects variable and trial type (pattern vs. random) and block (1−10) as within-subjects variables. Dependent measures in the ASRT were both Median reaction time and Percentage trials correct.

### 2.3. Materials

The tests were designed in Adobe Flash Professional CC, with a PHP back-end for client-server communication. The server hosting the web application was maintained by the IT-department at the University of Bergen. The study was conducted over the internet, but client-server communication was limited to initialization of each session, pauses, and session end. All critical variables and randomized aspects of the tests were constructed in advance by the server.

### 2.4. Procedure

Each participant conducted both tasks in the same order: First the sugar factory task, followed by the ASRT task. The study was conducted online, on computers of participants' own choice, and at a chosen time of day. However, participants were encouraged to conduct in the afternoon when they felt awake. All information and instructions were written in Norwegian.

#### 2.4.1. The sugar factory task

**Figure 1:**
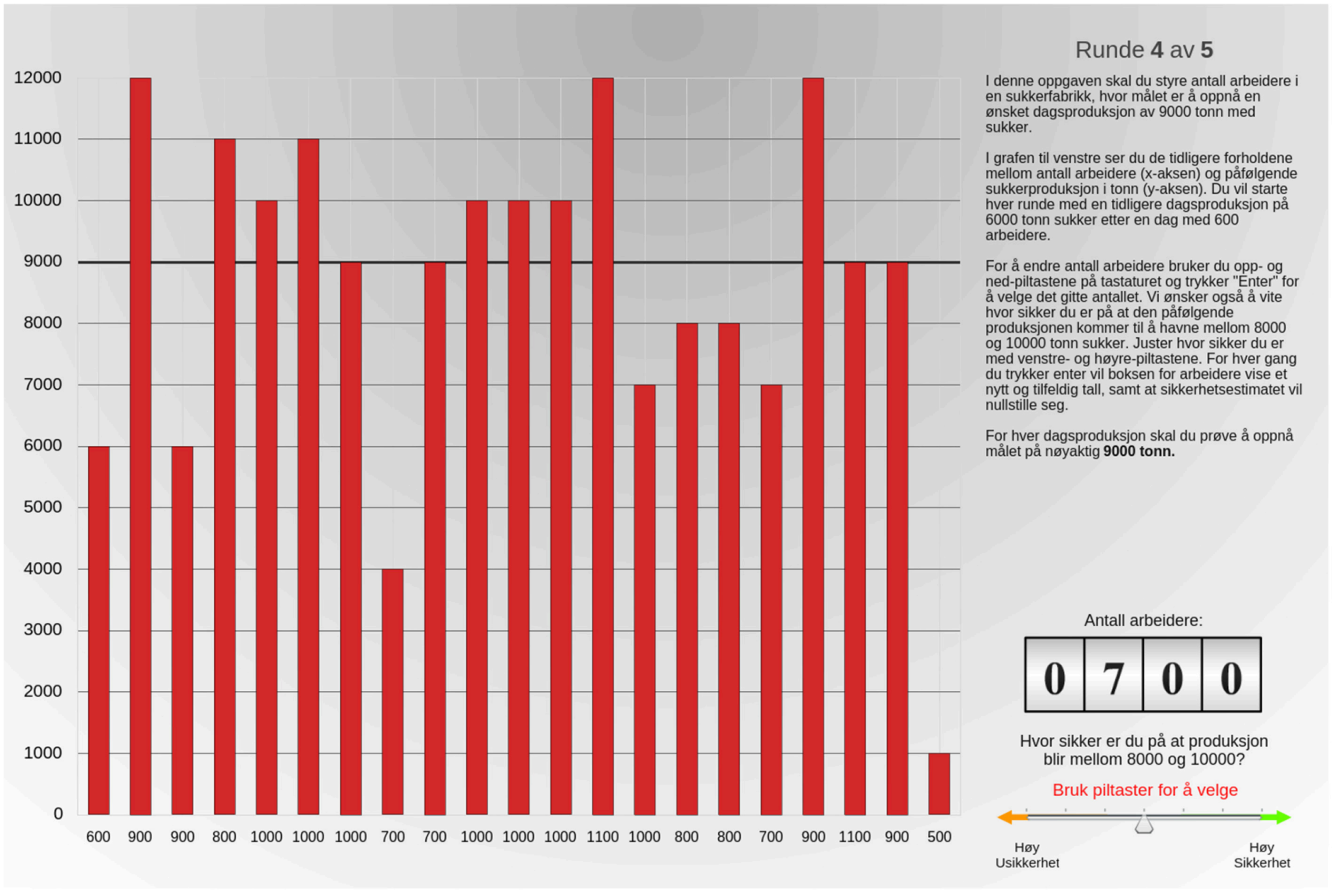
**A screenshot of the sugar factory task**. The block has here been finished.

In the sugar factory task, participants were instructed to imagine that they were in charge of a simulated sugar factory. More specifically, their task was to attempt to reach a target production value of 9000 ton sugar each simulated day (trial) by changing the workforce (Berry and Broadbent, 1984). The visual design of the task was based on Witt et al. (2006) and can be seen in Figure 1. To indicate the amount of workers, participants could press the up and down arrows on their keyboard to change the value of a counter. The worker input changed stepwise by 100, ranging from 100 to 1200, in addition to being circular, where an increment to the last input (1200) would lead to the first input (100), and vice versa. The algorithm controlling production value (*P*) as a function of the worker input (W) was *P*_*t*_ = 20^*^W_*t*_ − *P*_*t* − 1_ + *error* (Berry and Broadbent, 1984), with a minimum level of 1000 and a maximum limit of 12000. In line with previous implementations of the task, a random error of +1000, 0, or −1000 was used to reduce salience of the relationship. A diagram was shown throughout the task, where the vertical axis indicated level of production in tons, and the horizontal axis showed the worker inputs and rising bars for each consecutive trial. The diagram initially showed one previous production of 6000 tons of sugar, with a workforce of 600. The target production level of 9000 ton was made more salient by a bold black horizontal line. Before each trial, the starting value of the workforce counter was randomized.

A measure of subjective confidence was also included to assess the extent to which performance was based on conscious knowledge (Norman and Price, 2015). The confidence instructions asked participants to indicate the extent to which they felt confident that the production value would fall between 8000 and 10000. The confidence input was shown as a six-point horizontal slider with description ranging from left-most “Inconfident” to right-most “Confident,” divided into interim degrees of “High,” “Medium,” and “Low,” The confidence rating could be changed by pressing the left and right arrow on the keyboard. A specified level of confidence was required before the next production value would be shown, and with each trial the slider would return to the blank state. To complete a trial one would have to press “Enter” on the keyboard. An interval of 2 s was required between each trial, during which inputs were disabled. The graph animated the recently added production after each trial as a rising bar and the previous work force used was displayed beneath each respective bar.

The task was administered in 5 blocks. For each block, the graph would return to the initial state and previous trials would no longer be visible. Participants completed 20 trials on each block, but due to an error in the script communicating with the server, only the first 19 trials were recorded.

#### 2.4.2. Alternating serial reaction time task

**Figure 2:**
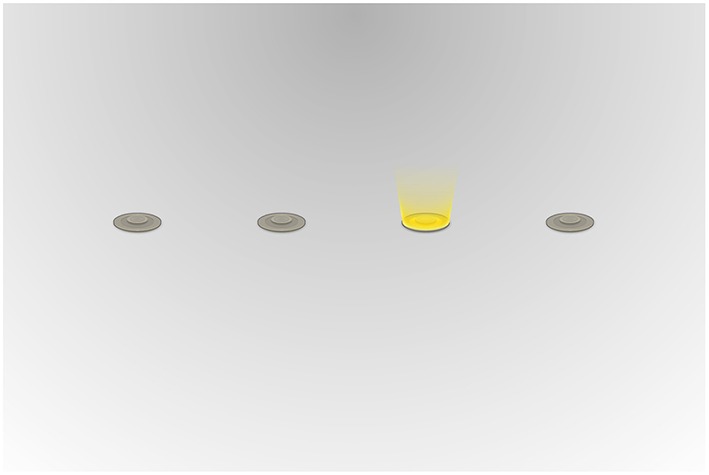
**A screenshot of the alternating serial reaction time task**. The spatial goal position for the next trial is highlighted with the yellow lighting. (“Small world” art by Daniel Cook; Lostgarden.com)

Throughout the alternating serial reaction time (ASRT) task, four circles placed evenly along a horizontal axis, were displayed on screen. On each trial, one of these circles was highlighted with a yellow beam radiating out from the circle (see Figure 2). This was the target stimulus. As soon as the target appeared, participants were to indicate its position by pressing a corresponding key on the keyboard. The response keys (corresponding to the target positions from left to right) were F, G, J, and K. Instructions encouraged participants to respond as quickly and accurately as possible on every trial. Correct responses elicited a green beam from the circle and a brief click sound, while incorrect responses elicited a red beam from the incorrect circle and a noisy beep. Both correct and incorrect responses would initiate the next trial and a new circle would light up. There was no measurable response-stimulus interval, and the next trial would start as the previous ended. The pattern of circles to be highlighted followed a repeating sequence, 4*r*3*r*2*r*1*r*, of which the numbers refer to their respective spatial location on the screen numbered from left to right and the letter *r* indicates a random spatial location (Howard and Howard, 1997). Every participant was exposed to the same pattern, but the random trials were generated independently for each participant and block. The target occurred in each location with an equal frequency, and the random trials were randomly permuted within each block.

The ASRT task was administered in blocks separated by short (15 s) breaks. Each block started with eight random trials, followed by the repeating sequence for 200 trials. The initial position of the sequence was randomly selected for each block, and the starting trial could also be either type of trial, i.e., pattern or random. After each block of the Single session condition, and at the start of each session for participants in the Multiple session condition, participants were given a summary of their performance in the preceding block, indicated both in terms of amount of correct trials per second and percentage of trials correct.

#### 2.4.3. Generation task and questionnaire

After completing all trials of both the sugar factory task and the ASRT task, participants were redirected to a questionnaire. This consisted of several sections, presented individually without the possibility of reverting and editing previous answers. First, participants were asked to select one or more out of four possible aspects in the ASRT task that they thought best predicted subsequent target positions (Norman et al., 2011). The alternatives were (1) “The positions of the previous buttons that lit up,” (2) “Whether I pressed correct or incorrect on the previous button that lit up,” (3) “How many mistakes I had made overall,” and (4) “The positions were random,” In the next section the participants were required to score the assumed relative importance on a forced weighted-choice task. The requirement to continue was that 12 points were assigned to one or more possible aspects in respect to assumed importance. A counter dynamically registered the total number of points assigned.

To assess the extent to which participants were able to strategically control their sequence knowledge, all participants also conducted an inclusion and exclusion generation task (Destrebecqz and Cleeremans, 2001). Participants were first informed that the sequence of target positions followed a pre-determined pattern that also contained random elements. Under inclusion instructions, they were encouraged to generate a series of 80 trials that would be as similar as possible to the pattern they had responded to previously in the tasks. They were told to follow their intuition when in doubt or unsure about the pattern. Under exclusion instructions, participants were told to create a new sequence of 80 trials in which they avoided generating the learned pattern. In both cases, participants were asked to avoid repeating longer sequences when generating. A restriction on the input was implemented but not mentioned in the instructions, namely that subsequent repetitions of the same position could not exceed 3. Attempts to add more than three subsequent repetitions thus did not elicit any visual or auditory feedback.

### 2.5. Sampling procedures

We first attempted to recruit participants by sending invitations to a large number of students by email, but due to a low response rate combined with a high mortality rate, we instead decided to recruit participants in undergraduate lectures with consequent snowballing among the students. Students recruited at the university campus were equally represented by 30 in each condition. From the participants recruited online, four were in the multiple sessions condition, and two were in the single session condition. The final sample size was *n* = 66.

## 3. Results

### 3.1. Performance on the two tasks

All analyses were conducted in R version 3.2.4 (R Core Team, 2016), using the package *ez* version 4.3 (Lawrence, 2015). ANOVA Sum of Squares type III was used as interaction effects were expected. Mauchly's test of sphericity were conducted on within-subjects variables. When significant and assumption of sphericity was violated, Greenhouse-Geisser estimate of sphericity were used to adjust degrees of freedom and are reported where relevant. Data from participants who dropped out before completing the whole test procedure were not included in any of the analyses. Exclusion of participants from analyses was done independently for the sugar factory task and the ASRT task, and the intersection of non-excluded participants were used for the cross-task comparison.

#### 3.1.1. Sugar factory task

Data were analysed using a mixed-design ANOVA with Block (1–5) as a within-subjects variable and Session (Single session vs. Multiple session) as a between-subjects variable. Both dependent measures, Number of trials on target and Mean confidence, were calculated independently for each block. Mauchlys test for sphericity was not significant for Number of trials on target, but significant for Mean confidence.

##### 3.1.1.1. Data reduction

Results were screened for response bias due to the possibility of responding without adjusting the randomized worker input of each trial.

Due to the circular adjustment method from the endpoints of the worker input, the amount of change from the randomized value to the one selected on each trial was calculated for both directions. E.g., a change from an initial randomized worker input value of 1200 to 100 could be calculated as a change of both 1 and 11, but trial-based change of least distance was selected.

The largest difference between two values on the circular scale from 1 to 12, in the direction of least distance, would therefore be 6. If worker input variables were selected independently from the randomized value for each trial, a mean change of three would be expected. It is reasonable to assume a small influence of the random variable on the worker input chosen due to the possibility of both an anchor effect and participants hypothesizing a predictive value. As the possibility and influence of such effects were not of interest for the current study, the exclusion criterion was rather strict. The possibility of excluding someone following instructions was preferred to including someone overly affected by the random variable. The exclusion criterion used to remove participants was therefore a mean change on the worker input of < 2. A total of 13 participants were excluded; eight from the single session condition and five from the multiple sessions condition, leaving *n* = 53 participants for the analysis of trials on target.

Because analyses of confidence were based on a comparison between trials that were on/off target, participants with blocks where none of the trials were within the target limits were excluded case-wise in addition to those excluded from the first analysis. Of those not already excluded, 10 participants met this criterion, leaving *n* = 46 participants for the analysis of confidence.

##### 3.1.1.2. Trials on target

**Figure 3:**
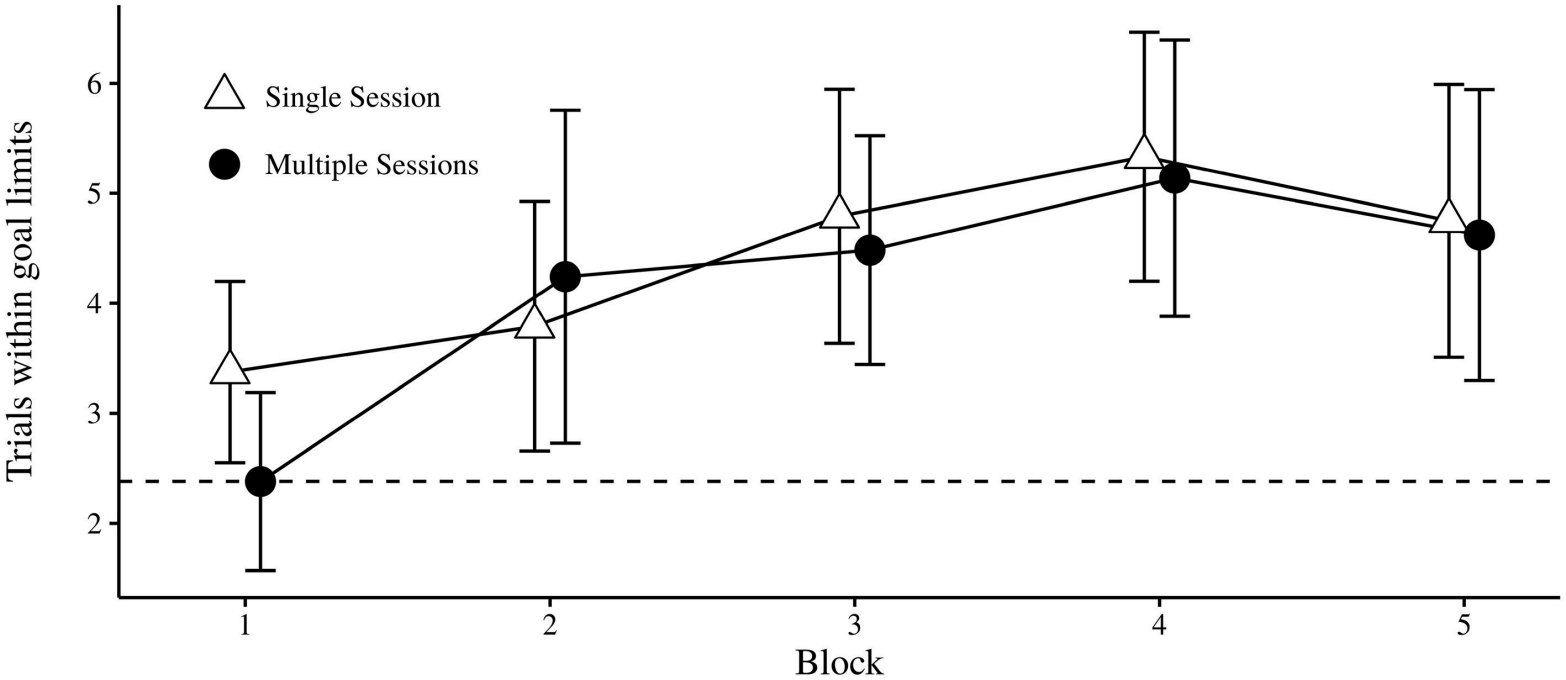
**Mean number of trials on target for each condition over successive blocks**. Error bars indicate 95% CI. The dotted horizontal line represents chance level.

Results from the analysis are shown in Table 1. Trials were dichotomized according to whether or not they were on Target (i.e., within a target production level of 8000−10000). There was no significant main effect of Session. The main effect of Block was significant, and a post-hoc analysis (Tukey's HSD test) showed that accuracy was higher in the last three blocks (*M* > 4.26) compared to the first (*M* = 2.89, *p* < 0.05). The Block × Session interaction was not significant. This indicates that there was no advantage of intermittent administration of the sugar factory task. The likelihood of achieving the target production level (i.e., within target limits) by random responding was 2.38. Chance level was calculated from a mean of 10, 000 simulations of 19 trials, with randomized worker input on each trial. Performance was generally better than chance level (see Figure 3).

**Table 1:** **Analysis of variance for sugar factory task with trials on target as dependent measure**.

**Effect**	**Mauchly's**	**GGϵ**	**df**	**df_*error*_**	***F***	***p***	**$\eta_{G}^{2}$**	**MSE**
Session			1	51	0.26	0.61	0.00	13.97
Block	χ^2^(9) = 15		4	204	5.79	< 0.001	0.07	7.36
*S* × *B*	χ^2^(9) = 15		4	204	0.48	0.75	0.01	7.36

*GGϵ, Greenhouse-Geisser correction epsilon. None of the Mauchly's tests of sphericity were significant (p > 0.05)*.

##### 3.1.1.3. Confidence

**Figure 4:**
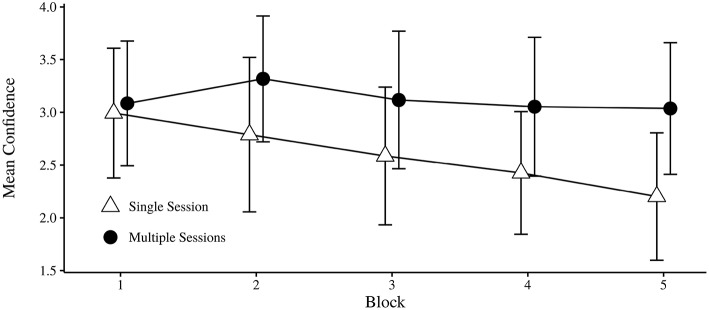
**Mean confidence for each condition over successive blocks**. Error bars indicate 95% CI.

The dichotomization of the trials as on/off Target was included as a independent variable for analysis of confidence. Neither the main effect of Session or Target was significant, nor was their interaction, as shown in Table 2. The main effect of Block was significant, and the interaction effect of Block × Session was not significant. However, the the confidence change over successive Blocks seems to be mostly accounted for by the Single session condition (see Figure 4). The interaction effect of Block × Target was not significant, indicating that the confidence did not seem to change with practice for trials that were on/off target. The final interaction effect of Session × Block × Target was not significant.

**Table 2:** **Analysis of variance for sugar factory task with confidence as dependent measure**.

**Effect**	**Mauchly's**	**GGϵ**	**df**	**df_*error*_**	***F***	***p***	**$\eta_{G}^{2}$**	**MSE**
Session			1	41	1.73	0.2	0.03	16.8
Block	χ^2^(9) = 22		4	164	3.75	0.006	0.01	0.8
Target			1	41	0.69	0.41	0.00	0.13
*S* × *B*	χ^2^(9) = 22		4	164	1.94	0.11	0.01	0.80
*S* × *T*			1	41	0.08	0.78	0.00	0.13
*B* × *T*	χ^2^(9) = 42	0.68	2.73	112.05	0.66	0.56	0.00	0.19
*S* × *B* × *T*	χ^2^(9) = 42	0.68	2.73	112.05	0.54	0.63	0.00	0.19

*GGϵ, Greenhouse-Geisser correction epsilon. Mauchly's tests of sphericity was significant (p > 0.05) for effects where GGϵ is reported*.

##### 3.1.1.4. Conscious knowledge

To examine whether performance was affected by whether one had conscious knowledge of the task regularity (cf. the zero-correlation criterion; Dienes et al., 1995), we compared the confidence of trials on/off target independently for each participant by applying Welch's unequal variances *t*-test (one-way). Participants who were significantly (*p* < 0.05) more confident for On Target trials as compared to Off Target trials were assumed to be at least partly aware of the learned regularity. Note that the effect of practice with increasing number of Blocks were not taken into account here, and exclusion should therefore not affect any effect differently. A total of nine participants met this criterion, of which three was already excluded due to response bias. We redid the analysis on trials On Target, and the overall findings did not change. All effects found to be significant were still significant when participants with possible conscious knowledge were excluded.

#### 3.1.2. ASRT task

Data were analysed using a mixed-design ANOVA with Block (1–10) and Trial Type (Random vs. Pattern) as within-subjects variables and Session (Single session vs. Multiple session) as a between-subjects variable. Trial Type was classified on the basis of the repeating sequence (4*r*3*r*2*r*1*r*), where the reoccurring fixed pattern positions 4, 3, 2, and 1 is pattern type trials. Every second trial, here represented as “*r*,” would be a random position, of which each possible position is uniformly distributed across each block. Both dependent measures, median reaction time (RT) of correct trials, and percentage of trials correct, were calculated independently for random and pattern trials for each block. Mauchlys test indicated that the assumption of sphericity had been violated for the within-subjects variable Block on both dependent measures; degrees of freedom were adjusted accordingly by applying Greenhouse-Geisser estimates of sphericity.

##### 3.1.2.1. Data reduction

As the target could always occur in any of the four target positions, the chance accuracy level would be 25% trials correct. As the task would continue to the next trial with wrong input, possible task negligence could manifest itself in a higher frequency of wrong responses. We also assumed negligent performance to be more prevalent with the online test setup, as participants degree of perceived monitoring would presumably be lower than in a traditional laboratory assessment. Results were screened for response bias, where an exclusion criterion of less than 75% correct trials within one or more blocks was applied. A total of nine participants were excluded due to response bias within the ASRT, whereof seven were single session participants and two were multiple session participants. The participants included in both analyses of the ASRT task and analysis of generation performance were thus *n* = 57.

##### 3.1.2.2. Reaction time

**Figure 5:**
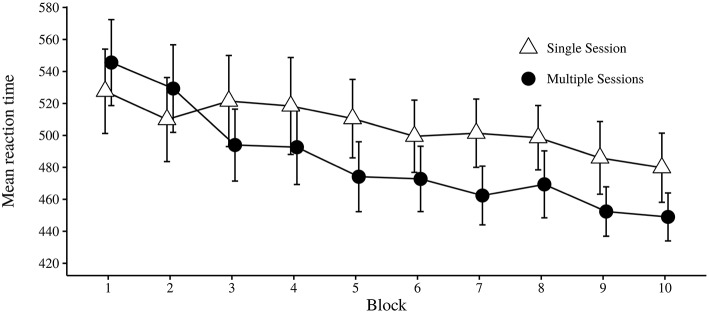
**Mean of median reaction time in milliseconds over each block, divided by single and multiple sessions conditions**. Error bars indicate 95% CI.

**Figure 6:**
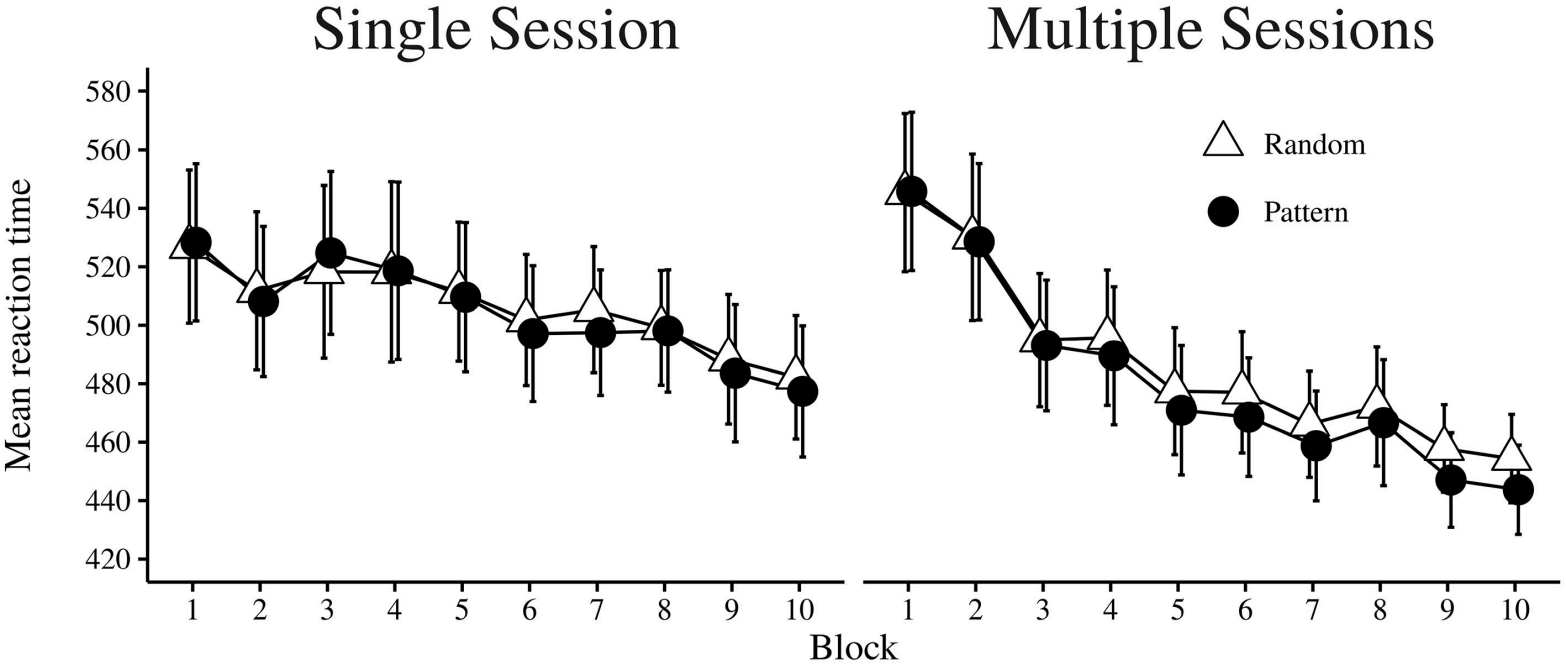
**Mean of median reaction time in milliseconds for pattern and random trial types, shown independently for each condition over each block**. Error bars indicate 95% CI.

The dependent measure of RT was the case-wise median of each Trial Type in each Block for correct trials (Howard and Howard, 1997). As shown in Table 3, the main effect of Session was not significant, indicating that the time condition did not have a consistent influence on differences in RT. However, numerically the overall RT was faster for participants in the multiple session condition (*M* = 484.16, *SD* = 66.66) as compared to the single session condition (*M* = 505.31, *SD* = 60.78). There was a significant main effect of Trial Type. Faster RTs were observed for pattern trials (*M* = 491.34, *SD* = 65.83) than random trials (*M* = 495.53, *SD* = 64.09). The interaction effect of Trial Type × Session was also significant. A post-hoc analysis (Tukey's HSD test) showed that in the multiple session condition, there was a significant difference between trial types (*p* < 0.001), with RT being faster for pattern trials (*M* = 481.23, *SD* = 67.45) than for random trials (*M* = 487.09, *SD* = 65.83). In the single session condition, there was no significant difference between pattern trials (*M* = 504.28, *SD* = 61.43) and random trials (*M* = 506.33, *SD* = 60.22, *p* = 0.35). The main effect of Block was significant, with a trend for reaction times to become faster with more practice (see Table 4). An interaction effect of Block × Session was also significant. As shown in Figure 5, the reduction in reaction time across blocks changed less for the single session condition than for the multiple session condition. The interaction of Block × Trial Type was significant. With increased practice there was a faster reaction time on pattern trials than for random trials. The last interaction effect of Block × Trial Type × Session was not significant. See Figure 6 for a graphical display of the relationship.

**Table 3:** **Analysis of variance for ASRT with reaction time (RT) as dependent measure**.

**Effect**	**Mauchly's**	**GGϵ**	**df**	**df_*error*_**	***F***	***p***	**$\eta_{G}^{2}$**	**MSE**
Session			1	55	2.04	0.16	0.03	61,651.81
Block	χ^2^(44) = 565	0.35	3.15	173	52.46	< 0.001	0.11	1091.12
Trial type			1	55	23.20	< 0.001	0.00	189.00
*S* × *B*	χ^2^(44) = 565	0.35	3.15	173	11.78	< 0.001	0.03	1091.12
*S* × *T*			1	55	5.42	0.02	0.00	189.00
*B* × *T*	χ^2^(44) = 227	0.81	7.30	401.62	4.34	< 0.001	0.00	83.54
*S* × *B* × *T*	χ^2^(44) = 227	0.81	7.30	401.62	0.92	0.49	0.00	83.54

*GGϵ, Greenhouse-Geisser correction epsilon. All Mauchly's tests of sphericity were significant (p < 0.05)*.

**Table 4:** **Descriptives for ASRT Reaction time for factors session, trial type, and block (1, 10)**.

	**Single session^a^**				**Multiple session^b^**			
	**Random**		**Pattern**		**Random**		**Pattern**	
**Block**	***M* (*SD*)**	**95% CI**	***M* (*SD*)**	**95% CI**	***M* (*SD*)**	**95% CI**	***M* (*SD*)**	**95% CI**
1	527 (63)	[501, 553]	528 (65)	[501, 555]	545 (75)	[518, 572]	546 (75)	[519, 573]
10	482 (51)	[461, 503]	477 (54)	[439, 469]	454 (42)	[439, 469]	444 (42)	[428, 459]

*CI, confidence interval. Due to limited space, only Block 1 and 10 are included*.

a *n = 25*.

b *n = 32*.

##### 3.1.2.3. Accuracy

**Figure 7:**
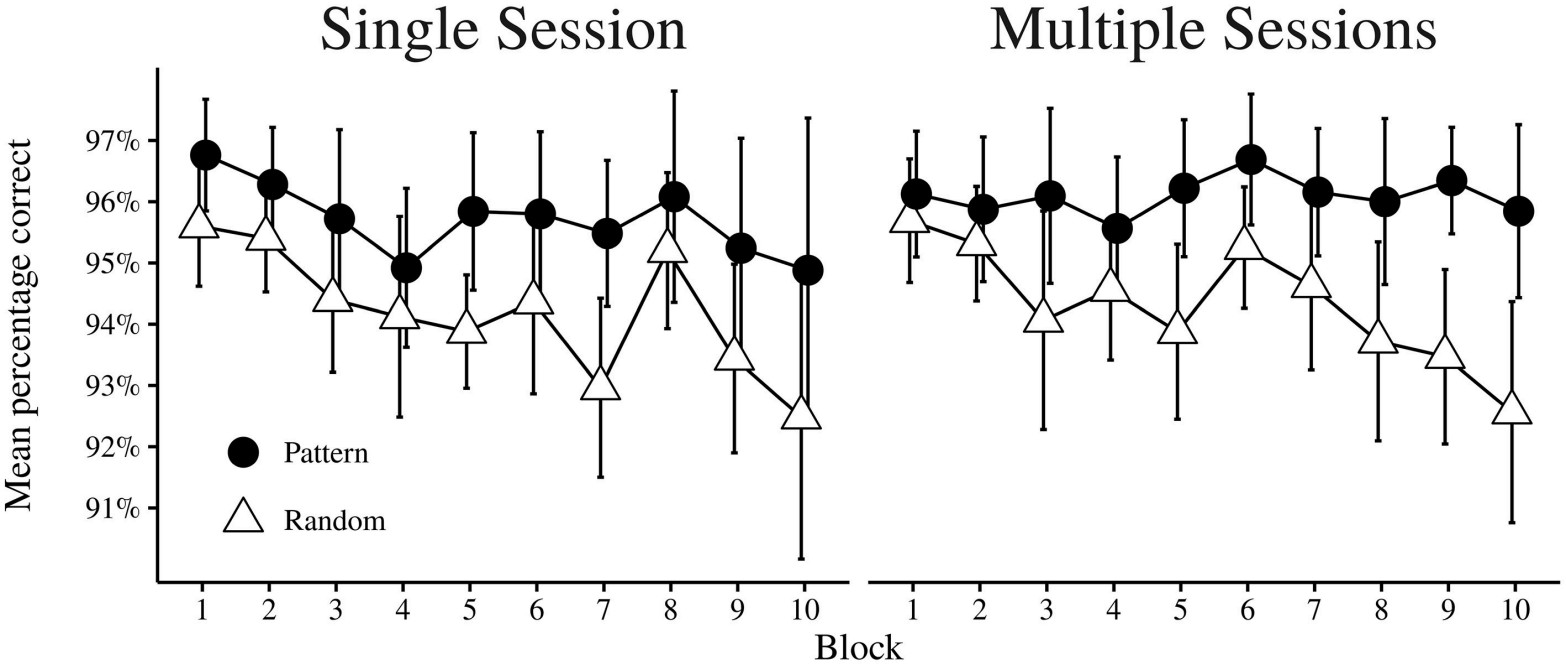
**Mean percentage correct trials of each block for pattern and random trial types, shown independently for each condition**. Error bars indicate 95% CI.

As shown in Table 5, the main effect of Session was not significant, and there was also no significant interaction between Session × Block, Session × Trial Type, or Session × Block × Trial Type. Thus, whether learning took place during a single session or across several sessions in the ASRT task seemed not to influence performance accuracy. However, there was a significant main effect of Trial Type. Participants were more accurate on pattern trials (*M* = 95.92%, *SD* = 3.42) than on random trials (*M* = 94.26%, *SD* = 3.74). The main effect of Block was also significant, with a trend for the proportion of correct trials to be reduced across subsequent blocks. The main effect of Block was however qualified by the Block × Trial Type interaction effect, since the reduction in proportion correct over Blocks was mostly accounted for by the random trial types (see Figure 7).

**Table 5:** **Analysis of variance for ASRT with proportion correct as dependent measure**.

**Effect**	**Mauchly's**	**GGϵ**	**df**	**df_*error*_**	***F***	***p***	**$\eta_{G}^{2}$**	**MSE**
Session			1	55	0.18	0.67	0.00	103.41
Block	χ^2^(44) = 120	0.64	5.75	316.33	3.50	0.003	0.03	11.53
Trial type			1	55	71.03	< 0.001	0.05	10.72
*S* × *B*	χ^2^(44) = 120	0.64	5.75	316.33	0.85	0.53	0.01	11.53
*S* × *T*			1	55	0.45	0.50	0.00	10.72
*B* × *T*	χ^2^(44) = 75	0.79	7.11	390.89	3.46	0.001	0.01	4.04
*S* × *B* × *T*	χ^2^(44) = 75	0.79	7.11	390.89	1.06	0.39	0.00	4.04

*GGϵ, Greenhouse-Geisser correction epsilon. All Mauchly's tests of sphericity were significant (p < 0.05)*.

##### 3.1.2.4. Generation performance

Data from both the inclusion and exclusion task were first reduced to triplets for analysis of performance. The 80 trials therefore led to 78 triplets for each generation task. Triplets were then coded as pattern consistent if they started and ended on a pattern type trial. Since the pattern was 4*r*3*r*2*r*1*r* (where *r* refers to any position), triplets like 4*r*3, 3*r*2, 2*r*1, and 1*r*4 would be coded as pattern consistent (Howard and Howard, 1997). All participants who completed the entire test procedure were included in the analysis. First, we tested if the proportion of pattern consistent triplets was higher than chance level. The performance level that would be achieved if input was completely random or not influenced by trial stimuli locations (e.g., if the person consistently pressed the same key), was 0.25 for pattern consistent triplets. Single sample *t*-tests (two-sided) were performed for each of the 4 combinations of condition and generation task. In all cells, there were more pattern consistent triplets than would be expected by random input (all *p*'s < 0.01). For an overview of the proportion of triplets observed in both generation tasks, the ASRT task, and triplets expected by chance, see Table 6.

**Table 6:** **Proportion pattern consistent triplets observed and expected by chance**.

	**Random span (%)**	**Random inconsistent (%)**	**Random repetition (%)**	**Pattern consistent (%)**
Inclusion task	9.2	56.5	3.4	30.9
Exclusion task	11.8	54.4	3.8	30.0
ASRT task	9.5	26.5	3.0	61.0^a^
Random input	18.8	50.0	6.2	25.0

*Triplet names are based on Howard and Howard (1997). Random span triplets start and end with the same trial position. Random Repetition triplets contain three consecutive repetitions of the same trial position. Pattern consistent triplets includes both actual pattern triplets, and random triplets by coincidence consistent with the pattern*.

a *By occurrence of triplet type: Random consistent triplets = 13.3% , Pattern triplets = 47.6%*.

**Figure 8:**
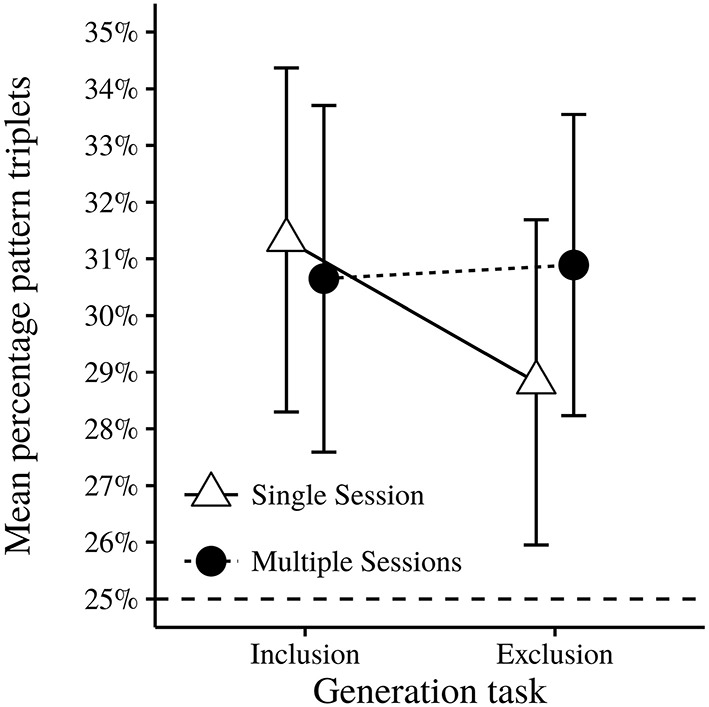
**Mean proportion of pattern type trials for each session type on both generation tasks**. Error bars indicate 95% CI.

The main analysis for the generation tasks performance was a 2 (Session) × 2 (Generation task) factorial ANOVA, using proportion of pattern consistent triplets as the dependent measure. The results of the analysis are shown in Table 7. None of the effects were significant (*p* > 0.05). The interaction effect of Session × Generation Task was not significant. However, there was a non-significant trend trend for participants in the Single session condition to generate less pattern consistent triplets in the Exclusion task (see Table 8) than in the Inclusion task, which could be taken to indicate strategic control. There was no such trend in the Multiple session condition (see Figure 8).

**Table 7:** **Analysis of variance for ASRT generation tasks with proportion pattern trials as dependent variable**.

**Effect**	***df***	***df_*error*_***	***F***	***p***	**$\eta_{G}^{2}$**	**MSE**
Session	1	55	0.16	0.70	0.00	0.009
Generation task	1	55	1.24	0.27	0.01	0.003
Session × Generation task	1	55	1.82	0.18	0.01	0.003

**Table 8:** **Proportion of generated pattern type trials in single and multiple session conditions**.

**Generation task**	**Single session^a^**			**Multiple session^b^**	
	***M* (*SD*)**	**95% CI**		***M* (*SD*)**	**95% CI**
Inclusion	0.31 (0.07)	[0.28, 0.34]		0.31 (0.09)	[0.28, 0.34]
Exclusion	0.29 (0.07)	[0.26, 0.32]		0.31 (0.07)	[0.28, 0.34]

*CI, confidence interval*.

a *n = 25*.

b *n = 32*.

##### 3.1.2.5. Conscious knowledge

As in the sugar factory task, it is possible that the results could be explained in terms of conscious sequence knowledge, indicating that learning may have been explicit rather than implicit. Participants with possible conscious knowledge of the task were thus excluded on the basis of response on the questionnaire. In the forced weighted-choice task of the four possible aspects of the sequential nature of the ASRT task, where 12 points had to be assigned to indicate assumed importance, participants with a score of five or more on the importance of “position of previous trials” were excluded. Six participants met this criterion (two single session, four multiple session). We redid the three analyses mentioned above on the ASRT task and Generation tasks, and all previously significant effects remained significant in the new analyses, suggesting that the reported effects were not limited to participants with explicit sequence knowledge.

### 3.2. Correlation of implicit learning between tasks

Comparison of the performance in the two tasks was done using of the same measures as those used by Gebauer and Mackintosh (2007), though differently coded. The measure of interest in the ASRT task was the individual ratio of RT for Random type trials to Pattern type trials (i.e., median RT for Random divided by median RT for Pattern) in the last block. Gebauer and Mackintosh originally used RT difference, but this absolute measure does not account for the large individual variation in overall RT. In the sugar factory task the measure used was the mean number of trials On Target across all Blocks. Participants previously excluded due to response bias in either tasks were also excluded for the analysis of task performance correlation, leaving *n* = 48. The Pearson correlation coeffecient between the two measures was not significant, *r*_(46)_ = 0.19, *p* = 0.21.

Another index of the degree of learning on the ASRT task would be the individual cumulative difference of RT between Random type trials and Pattern type trials across all blocks. Using this measure for the ASRT, the Pearson correlation coeffecient between the two measures was also not significant *r*_(46)_ = 0.14, *p* = 0.36.

Performance on implicit learning tasks such as the ASRT task is often characterized by an increased RT difference on random trials compared to pattern trials from one block to the next. We therefore also calculated this cumulative relative block difference of RT ratio (the ratio of RT for random type trials to pattern type trials) difference across successive blocks for both tasks. The cumulative difference of the ratio was used as the performance improvement measure. To calculate the improved performance in the DSC task, the cumulative difference in blockwise trials on target was used. The Pearson correlation coeffecient was not significant, *r*_(46)_ = −0.03, *p* = 0.83.

The number of participants who, according to our criteria, had conscious knowledge were nine for the sugar production task, and six for the ASRT task. The intersection of participants with possible conscious knowledge, e.g., participants who met the criteria for both tasks, was one participant. Meeting the criterion for conscious knowledge for one task was not predictive of conscious knowledge in the other tasks.

### 3.3. Time usage

The total time spent on the tasks was dependent on the individual's performance, where fast responses on each trial would end the experiment earlier. In principle, differences between conditions in performance, motivation, and fatigue would therefore affect the total time spent. The description of time usage includes participants not excluded due to response bias for the respective task, and either task for the calculation total time. For the single session condition, the mean time spent on one block in the DSC task was 4.13 min (*SD* = 2.12, *skew* = 1.33, *n* = 100), with a total DSC task mean time of 20.2 min (*SD* = 5.59, *skew* = −0.1, *n* = 24). The time spent on one block in the ASRT task was 2.06 min (*SD* = 0.28, *skew* = 2.34, *n* = 250), with a total ASRT task mean time of 20.6 min (*SD* = 2.31, *skew* = 0.25, *n* = 25). The total time spent on both tasks for the single session condition thus averaged 40.96 min (*SD* = 6.93, *skew* = −0.2, *n* = 20). For the multiple sessions condition, the mean time spent on one block of the DSC task was 4.94 min(*SD* = 3.57, *skew* = 1.95, *n* = 140), with a total DSC task mean time of 24.24 (*SD* = 10.44, *skew* = 1.33, *n* = 29). The time spent on one block of the ASRT task was 1.86 min(*SD* = 0.29, *skew* = 1.47, *n* = 320), with a total ASRT task mean time of 18.64 min (*SD* = 2.16, *skew* = 1.6, *n* = 32). The total time spent on both tasks summed together for participants in the multiple sessions condition averaged 43.39 (*SD* = 10.44, *skew* = 1.21, *n* = 28).

Due to the differences between conditions in time spent on both tasks, we decided to do a correlation of total task duration and task performance measures of those participants who did not show explicit learning, as it could potentially explain group differences found in our previously mentioned findings. For the ASRT task, time spent on the task was correlated with the individual ratio of RT in Random type trials to Pattern type trials in the last block used previously. There was a non-significant trend for total time spent on the ASRT task and the RT difference between random and pattern trials (*r* = −0.26, *p* = 0.07). However, the correlation trend was mainly caused by participants in the Single session condition, as the same correlation done independently for each conditions showed almost no correlation for Multiple session (*r* = −0.07, *p* = 0.7), while the Single session condition correlation was stronger (*r* = −0.38, *p* = 0.07), although insignificant. We also did an additional correlational analysis to check if ASRT task total time could have affected generation performance, calculated as ratio of pattern consistent triplets in inclusion to exclusion task, which was not significant (*r* = −0.22, *p* = 0.31).

There was no significant correlation for the DSC task when the total task time was correlated with total trials on target, both overall and condition-wise (all *p*'s > 0.7).

## 4. Discussion

The study was designed to explore between-subject differences in learning when exposure to the regularity was intermittent over successive days as opposed to a continuous exposure (multiple sessions vs. single session). The tasks used were both the Dynamic Systems Control task of sugar factory (DSC; Berry and Broadbent, 1984) and the Alternating Serial Reaction Time task (ASRT; Howard and Howard, 1997).

The results from the sugar factory task indicated that learning occurred, and that participants' ability to control performance became increasingly better with more training. Learning was however not significantly affected by condition. Moreover, confidence was not significantly related to accuracy, which indicates that performance was unlikely to be mediated by conscious knowledge of the learned regularity. This was confirmed by the fact that results remained the same also when we excluded participants who were significantly more confident for on target than off target trials.

Learning also occurred in the ASRT task. This was indicated by reduced RTs across successive blocks, faster RTs for trials that followed the pattern compared to random trials, as well as RTs for pattern type trials becoming increasingly better than random type trials with more practice. Learning as shown by proportion correct was also found in the ASRT task, where accuracy was better for pattern type trials than random type trials. This divergence in accuracy between the two trial types also significantly increased across successive blocks, where accuracy for pattern type trials was relatively stable from start to end and accuracy for random type trials became lower with practice. On the ASRT task there was an effect of condition, in that multiple session participants showed more learning, both measured by RT across time and RT differences between the two types of trials. However, the two groups did not differ in terms of accuracy as a measure of learning. Moreover, there were no significant group differences in generation performance under inclusion or exclusion instructions. Both groups generated significantly more pattern consistent triplets in both generation tasks than one would expect from chance. Automatic application of the learned regularity in the exclusion task supports the assumption that the learning was implicit (Jacoby, 1991).

### 4.1. Relationship between implicit learning in different tasks

If implicit learning as measured by different tasks are expressions of the same learning mechanism, a relationship between different tasks measuring implicit learning would be expected. The literature to date suggests that the relationship between tasks is non-existent or negligible (e.g., data from Gebauer and Mackintosh, 2007). The two measures correlated in the current study was the RT ratio of random to pattern type trial in the ASRT task, and the mean trials On Target per block in the sugar factory task. The correlation was positive, but not significant. One should however note that the correlation found by Gebauer and Mackintosh (2007), using other variations of the same measures, was *r* = 0.01 and non-significant, while the non-significant correlation in the current study was stronger (*r* = 0.19).

There might be several reasons why the current study did not find any clear relationship between the two tasks. The most obvious is that there is no relationship, or that it is negligible. Because performance on the DSC task was compared to ASRT (rather than SRT) performance, the likelihood that the lack of a systematic relationship between performance on the two tasks is due to one task reflecting more motor learning and the other reflecting abstract learning of higher-order structures, is reduced. Thus, our findings could be seen to corroborate the findings of Gebauer and Mackintosh, who argued that different implicit learning tasks do not necessarily reflect a unitary ability. They are also compatible with the findings by Pretz et al. (2010), who found that performance on an SRT task and an AGL task were not systematically related. Pretz et al. (2010) argued that this may be related to the SRT being more “implicit” than the AGL task. However, this argument is unlikely to be applicable to our study, since we found that the proportion of participants who showed conscious awareness of acquired knowledge (which could be indicative of explicit learning) was comparable across the ASRT and DSC tasks.

It is also possible that the tasks measure different kinds of learning without conscious awareness. While the ASRT task requires fast motor responses and sustained perceptual vigilance, the sugar factory task does not and is arguably more cognitive in nature. It is thus possible that the tasks recruits different learning and memory systems. Comparisons across tasks that require the same degree of motor involvement would avoid this possible confound, and should therefore be considered for future studies that aim to assess whether different implicit learning tasks involve similar cognitive processes and/or individual differences in implicit learning.

Another important aspect to consider is the fact that any measure of learning, including reaction times in the ASRT task and worker input in the DSC task, are only approximates of learning, which may not necessarily reflect learning per se. In addition, reaction times are also prone to vary over time due to motivational factors, distractions, and shifts in attention. Both tasks are also dependent on the participants' motivation to perform, the attention directed to both the visual stimuli task feedback, as well as participants' choice of task strategy, all of which may vary over trials and time. This may in turn reduce the reliability of the measures.

On the DSC task, nine participants were classified as responding on the basis of conscious knowledge because they were significantly more confident on correct than incorrect trials. For the ASRT task, the number of participants expressing conscious knowledge of the sequence, as indicated by allocating five or more points to the correct dimension, was six. Only one participants showed conscious knowledge according to both these criteria. On both tasks, performance was not affected by removing those participants classified as being aware of either of the two regularities, which indicates that successful responding did not solely depend on conscious knowledge. In addition, exclusion performance on the ASRT task was above chance, which indicates that participants did not have full conscious control over sequence knowledge. Together, the overall results indicated comparable levels of conscious awareness on the two tasks even though performance on the two tasks did not correlate.

### 4.2. Intermittent exposure to implicit learning tasks across several sessions

Another theoretical question addressed in the current study was whether intermittent administration of implicit learning tasks would affect performance relative to a traditional single session administration. In the sugar factory task, performance was measured as number of trials where the target sugar production was reached. The multiple and single session conditions did not differ in how many trials were on target. The two groups were also equivalent in performance improvement with task practice. A non-significant trend did however indicate that the conditions differed in reported confidence over successive blocks, where participants in the Single Session condition showed reduced confidence with practice. However, self-reported confidence was not related to the accuracy on group level. One way to explain reduced confidence over successive blocks in Single session participants might be that there are differences in the availability of metacognitive evaluations of performance. In single session participants, confidence is likely to be influenced by metacognitive monitoring of performance on previous blocks, at least in part. However, in multiple session participants, such feelings are likely to be less salient, simply because the tests are distributed over several days.

For the ASRT task, the intermittent exposure could in principle relieve possible fatigue-effect. For the dependent measure of RT, the groups did perform differently on the ASRT task both over successive blocks and as a function of trial type. The increasing speed in RT over successive blocks was higher in the Multiple session condition, though it seems like the RTs in the first session (block 1 and 2) were indicative of a relatively cautious approach to the task as compared to the same RTs in Single Session (see Figure 5). Note also that the difference in RT between the first and the third block in the Multiple Sessions condition (*M* = 51.6), which is the first block of the first two sessions, was approximately the same as the difference in RT from the first block to the last block in the Single session condition (*M* = 47.82). If the participants in the multiple session condition approached the ASRT task more cautiously, one would also expect a relatively higher accuracy as compared to the single session condition in the same first two blocks. The combined accuracy in the first two blocks for each condition was therefore compared in a Welch two-sample *t*-test (two-tailed). There was no significant difference in the combined RT of the first two blocks for multiple session (*M* = 95.75) and single session conditions (*M* = 96.01); *t*_(54.98)_ = 0.50, *p* = 0.62. Even though this group difference in RT between the first two blocks may have been coincidental, it is also compatible with the idea that the two groups may have chosen different task strategies. The relative improvement from the first to the third block in the Multiple Session condition compared to the Single Session condition could also be explained in a number of different ways. More specifically, it could be due to (1) a memory consolidation of learned regularity after sleep, (2) improved learning due to a more cautious task strategy, (3) a change in strategy, or (4) a combination of the above. It is also possible that the relatively low improvement of RT performance observed for the Single Session condition is due to a early appearing fatigue-effect. Future studies should investigate whether this tendency could be explained by different strategies and approaches to the task.

Our analysis indicated that a small subset of participants showed evidence of conscious knowledge. For the DSC task, those who were significantly more confident for trials on target as compared to trials off target were categorized as potentially having conscious knowledge of the regularity (*n* = 9). In the ASRT task, those who rated “position previous trials” with five or more on the questionnaire, were categorized as potentially having conscious sequence knowledge (*n* = 6). To determine whether the proportion of participants who met ether of these criteria were unequally balanced across conditions, chi-square analyses were performed with Condition × Conscious knowledge criterion as categorical variables. We conducted three analyses. In two of these, each of the conscious knowledge criteria were applied, respectively. In the third analysis, the two criteria were combined, i.e., participants were defined as having conscious knowledge if both criteria were met. Neither of the chi-squares were significant (all *p*'s > 0.28). Participants were thus equally likely to have developed conscious knowledge in the two conditions, indicating that intermittent administration did not markedly affect the degree of conscious awareness.

A limitation of the current study was that we did not measure individual differences in circadian rythm, nor did we ask participants about their sleep pattern. Moreover, the time frame within which they could conduct individual sessions, was relatively wide. With respect to the time span between test sessions and circadian factors, Robertson et al. (2004) found that repeated exposure to the SRT task significantly improved performance for participants when the inter-session interval was 12 h as compared to an inter-session interval of 15 min. The improvement remained the same regardless of whether the time span included a period of sleep (8 p.m. vs. 8a.m.) or was without sleep (8a.m. vs. 8p.m.). The authors concluded that cross-session improvements should not be explained by repeated practice alone, but that off-line learning leads to greater improvements, which furthermore is relatively unaffected by circadian factors such as sleep. Circadian factors may however be related to performance as found in the study by Delpouve et al. (2014). The experiment indicated that implicit learning of higher order information in an AGL task was improved when the task was performed at individuals subjectively rated non-optimal time of day as compared to their optimal time of day. It could also be the case that sleep selectively influences the acquisition of certain types of knowledge in implicit learning experiments. Song and Cohen (2014) found that sleep improved the learning of a particular form of sequence knowledge, namely the ordinal positions of sequence elements. However, it did not influence learning of transitions between consecutive items, which was instead influenced by amount of practice. The relevance of including measures of sleep and circadian factors is also demonstrated by the findings of Fischer et al. (2006), who found that sleep facilitated the transition from implicit to explicit knowledge in sequence learning.

The proportion of correct trials in the ASRT task did not differ between the two conditions, either overall, as a function of practice with successive blocks, or as a function of trial type. When RT but not accuracy is affected by intermittent task exposure, this might again indicate different strategies or that fatigue affects the two measures differently. Our analysis also indicated that the ASRT task duration, when administered in a single session, could be related to implicit learning. Though the correlation was non-significant, participants who finished the task earlier tended to have a larger RT ratio of Random to Pattern type trial in the last block.

For the generation task, there was no significant difference in proportion of pattern consistent triplets generated as a function of task instruction. One should however note that there was a non-significant trend for participants in the Single session condition to show a reduced proportion of pattern consistent trials in the exclusion task than in the inclusion task. In the experiment done by Destrebecqz and Cleeremans (2001), participants with a 250 ms response-stimulus interval (RSI) performed better on the exclusion task as compared to participants in the no-RSI condition. Inspired by the process dissociation framework (Jacoby, 1991), they interpreted this in terms of knowledge being more conscious at higher RSI's. Thus, based on the non-significant trend observed in the current study one may speculate that knowledge was more conscious in the single session condition.

### 4.3. Online administration of implicit learning tasks

The overall results from each of the tasks applied in the current study do not deviate markedly from previous findings in laboratory experiments employing similar tasks (Gibson, 1996; Howard and Howard, 1997). Different levels of RTs on the two trial types of the ASRT tasks, and above-chance learning on the DSC task, both support the feasibility of implicit learning tests administered online. Based on our experiences from the current study, there are no major drawbacks to online administration of implicit learning tasks. However, one negative aspect of online data collection in general is that of the constricted form of communication, in which qualitative feedback from users is hard to follow up on. Our experience with online recruitment also seems to be disadvantageous as compared to traditional face-to-face recruitment, although it is not known whether this is due to characteristics of our sample, the fact that the study was conducted during the summer months, or the recruitment method itself.

### 4.4. Limitations

As the results from the current study is consistent with previous studies using the same tasks, we regard the design of the study and the means of administration as highly feasible. We do however acknowledge that there are aspects of the design which might potentially reduce the validity of the findings.

When recruiting participants to the study, we were required to inform potential participants about its duration. The main reason for this was to prevent mortality in the multiple session condition. Also, to counter the possible influence of demand characteristics caused by participants in the two conditions communicating about the conditional design, conditions were presented as independent studies. As we informed each group of potential participants of only one of the two conditions, a strict randomization of participants to the conditions was therefore not possible. The design of the study should thus be considered quasi-experimental. Though the sampling frame was the same for both conditions, different times of recruitment and individual preferences for both incentive and time requirement could have led to a selection bias. The results should be interpreted with this in consideration.

It is possible that the reliability of our measures may be reduced as a consequence of administering the tests in an uncontrolled environment. First, the setting and time was chosen by the participants, and distractions both on the computer and in the surroundings could thus vary for each participant, but also over time and across sessions for those in the multiple sessions condition. Second, the computers chosen by participants could also vary in both performance and screen refresh rate. The study also relied on the local time of each participants' computer for measuring RT, where scheduled automatic online synchronization of the time would make the RT of the concurrent trial incorrect. The use of median RT does however circumvent this potential flaw. Third, the test setup could not stop participants from taking an unforeseen break in the middle of performing a task, but the data did not suggest that this was a problem^2^.

ASRT tasks comparing pattern and random trial types as dependent variables usually expose participants to an extensive number of trials, often more than 10,000 (Howard and Howard, 1997; Howard et al., 2004), while the participant in the current study were only exposed to 2080 trials. It would however not be feasible increase the amount of trials, as the single session condition would last too long. Other studies on ASRT overcome the need for extensive exposure by comparing high- and low-frequency triplets (respectively pattern consistent to pattern inconsistent triplets), irrespective of whether the pattern consistent triplets occur by chance on random type trials (Nemeth and Janacsek, 2011; Nemeth et al., 2013).

## Ethics statement

The project was approved by the Norwegian Data Protection Official for Research, which is the Norwegian official for assessing whether research projects are conducted in accordance with the Personal Data Act and the Personal Health Data Filing System Act. The approval means that the study met the requirements for digital data collection and storage.

## Author contributions

This study was conducted within a student scholarship project granted to WS. The supervisor for this project was EN. Both authors contributed to the research design, data analysis, interpretation, and critical revision of the article. WS programmed the test setup, and had the main responsibility for data collection and handling, as well as drafting the paper.

## Funding

The project was supported by the Research Council of Norway (grant no. 163218; Framework Agreement for Student Research) and Skipsreder Jacob R. Olsen og hustru Johanne Georgine Olsens legat (grant no. 2015/9/FOL/BEKR).

### Conflict of interest statement

The authors declare that the research was conducted in the absence of any commercial or financial relationships that could be construed as a potential conflict of interest.

## Footnotes

^1^The role of motor learning in the SRT task has been the topic of much debate, but is a question beyond the scope of this paper, and will therefore not be discussed further.

^2^11 of the ASRT trials lasted longer than 5 s.
